# Increasing incidence of skin disorders in children? A comparison between 1987 and 2001

**DOI:** 10.1186/1471-5945-6-4

**Published:** 2006-03-21

**Authors:** Robbert SA Mohammedamin, Johannes C van der Wouden, Sander Koning, Michiel W van der Linden, François G Schellevis, Lisette WA van Suijlekom-Smit, Bart W Koes

**Affiliations:** 1 Department of General Practice, Room FF 304, Erasmus MC-University Medical Center Rotterdam, PO Box 1738, 3000 DR Rotterdam, The Netherlands; 2 NIVEL, Netherlands Institute for Health Services Research Utrecht, PO Box 1568, 3500 BN Utrecht, The Netherlands; 3 Department of Paediatrics, Erasmus MC-University Medical Center/ Sophia Children Hospital. Rotterdam, PO Box 2040, 3000 CA Rotterdam, The Netherlands

## Abstract

**Background:**

The increasing proportion of skin diseases encountered in general practice represents a substantial part of morbidity in children. Only limited information is available about the frequency of specific skin diseases. We aimed to compare incidence rates of skin diseases in children in general practice between 1987 and 2001.

**Methods:**

We used data on all children aged 0–17 years derived from two consecutive surveys performed in Dutch general practice in 1987 and 2001. Both surveys concerned a longitudinal registration of GP consultations over 12 months. Each disease episode was coded according to the International Classification of Primary Care. Incidence rates of separate skin diseases were calculated by dividing all new episodes for each distinct ICPC code by the average study population at risk. Data were stratified for socio-demographic characteristics.

**Results:**

The incidence rate of all skin diseases combined in general practice decreased between 1987 and 2001. Among infants the incidence rate increased. Girls presented more skin diseases to the GP. In the southern part of the Netherlands children consulted their GP more often for skin diseases compared to the northern part. Children of non-Western immigrants presented relatively more skin diseases to the GP. In general practice incidence rates of specific skin diseases such as impetigo, dermatophytosis and atopic dermatitis increased in 2001, whereas warts, contact dermatitis and skin injuries decreased.

**Conclusion:**

The overall incidence rate of all skin diseases combined in general practice decreased whereas the incidence rates of bacterial, mycotic and atopic skin diseases increased.

## Background

In general practice, skin disease accounts for a substantial part of morbidity in children and adolescents [1-4]. Compared to 1987, in 2001 the childhood morbidity encountered in Dutch general practice has changed; proportionally more skin diseases were presented to the general practitioner (GP) whereas other most frequent diseases (e.g. respiratory tract and general diseases) were presented less often. By the same token the overall consultation rate in general practice decreased by 22% [5,6]. Did the incidence rate of skin diseases in general practice increase?

However, little information is currently available about the epidemiology of skin diseases encountered in general practice. The few studies which have been performed show a wide variety in the occurrence of skin diseases presented to GPs [3,4,7]. Against the background of the changing consultation behaviour in general practice [5] and the increasing population-based prevalence of some skin diseases (e.g. atopic eczema) [8,9] it is important to estimate current incidence rates of the different skin diseases affecting children and adolescents in general practice. Further, primary care epidemiology can contribute to wider improvements in health and health care services, through better understanding of disease aetiology, use of health care services and the role of different health care interventions [10].

The present study relies on two consecutive surveys which were performed in Dutch general practice in 1987 and 2001. As they included all patient-physician contacts during a one-year study period, selection bias and the influence of seasonal variation are avoided.

To estimate current incidence rates of skin diseases affecting children and adolescents and to generate reference material for future studies, we conducted a detailed analysis of the skin diseases encountered in Dutch general practice between 1987 and 2001.

Our research questions were:

• How often did the GP see children aged 0–17 with specific skin diseases; to what extent did that change between 1987 and 2001?

• Was the incidence rate of skin diseases encountered in general practice in 1987 and 2001 related to socio-demographic characteristics?

## Methods

We used data from the first and second Dutch national surveys of general practice, which were performed by the Netherlands Institute for Health Services Research (NIVEL) in 1987 and 2001. In the Netherlands, general practices have a fixed list size, and all non-institutionalised inhabitants are listed in a general practice, and GPs have a gate-keeping role. Usually, the first contact with health care, in a broad sense, is the contact with the general practitioner. Each survey included a representative sample of the Dutch population.

In 1987 practices were randomly sampled from a list of all Dutch practices, per stratum defined by region and degree of urbanization. Sampling fractions differed between strata. 161 GPs in 103 practices participated in the first national survey [11]. With respect to age and gender the participating GPs and practices were representative of Dutch GPs and practices in 1987. The GPs were divided into four groups, and each group used registration forms to register data (e.g. diagnosis, prescription and referrals) on all contacts between patient and practice during one of four consecutive 3-month periods. Baseline characteristics such as age and gender were derived from patient records. Other socio-demographic characteristics such as socio-economic status (SES) and ethnicity were obtained by a questionnaire and filled out by parents, or by the children themselves if they were older than 12 years (response rate 91.2%). SES was based on the father's occupation, which was categorized into five classes "non-manual work high (class I)", "non-manual work middle (class II)", "non-manual low and farmers (class III)", "manual work high / middle (class IV)" and "manual work low (class V)". Ethnicity was derived from the country of birth of either parent. If either parent was born in Turkey, Africa, Asia (except Japan and Indonesia) and Central or South America, their children were considered to be children of non-Western origin (in accordance with the classification of Statistics Netherlands). All other children were defined as Western. The degree of urbanization was derived from the general practice's postal code and categorized into four classes 'under 30,000 inhabitants', '30,000–50,000 inhabitants', 'over 50,000 inhabitants' and 'the three large Dutch cities Amsterdam, Rotterdam and The Hague'. The Netherlands were divided into a Northern, Central and Southern region. Season was divided into four categories: spring was defined as months April-June, summer as July-September, autumn as October-November and winter as January-March.

The diagnoses made by the GPs were coded afterwards by clerks using the International Classification of Primary Care (ICPC) [12].

In 2001, 195 GPs in 104 practices registered data about all physician-patient contacts over 12 months [13]. They registered all health problems presented within a consultation, and coded the diagnosis themselves using the ICPC. Patient demographic characteristics such as age and gender were derived from the GP's computerized patient files. As in 1987, SES and ethnicity were obtained by a questionnaire (response rate 76%). Degree of urbanization, region and season were derived as in 1987.

In both surveys each contact with the GP was defined as one consultation. All health problems presented within one consultation were recorded separately. Both surveys were episode orientated, meaning that a consultation on a new health problem marked the beginning of a new episode. If there were multiple consultations in a single episode, the diagnosis made during the last consultation was regarded as the episode-diagnosis. In order to decide whether two consultations with the same problem belonged to the same episode or were different episodes, the latter was arbitrarily decided upon if the interval between two consultations was at least four weeks (28 days).

There were 20 practices that participated in both surveys. In 2001 eight practices were excluded from analyses for the following reasons: two practices had software problems; one practice registered only over a three-month period; five practices showed insufficient quality of the morbidity registration.

### Ethical approval

The study was carried out according to Dutch legislation on privacy. The privacy regulation of the study was approved by the Dutch Data Protection Authority. According to Dutch legislation, obtaining informed consent is not obligatory for observational studies.

### Statistical analysis

This study analyzed data from both surveys for children aged 0–17 years presenting with skin diseases, classified by ICPC codes. Incidence rates in general practice were calculated for all combined skin diseases and for each skin disease separately with a distinct ICPC code. We calculated the incidence rate by dividing the total number of new episodes (numerator) by the study population at risk multiplied by the follow-up time (denominator). In 1987 the denominator was calculated by multiplying the number of all patients listed in the participating practices by the follow-up time (person years). In 2001, persons that moved into or out of the participating practices during the registration period were assumed to contribute for half a year to the follow-up time. The so-called mid-time population was calculated as the mean of all listed patients of all participating GPs, aged 0–17 years, at the beginning and at the end of the registration period, irrespective of health care use. Data were stratified for age categories, gender, urbanization level, region, season, SES and ethnicity.

Further we assessed the changes in incidence rates of all skin diseases between 1987 and 2001. Incidence rates were expressed per 1000 person-years; 95% confidence intervals (CI) were calculated assuming a Poisson distribution. Skin diseases which contributed less than 0.5 percent to the total skin morbidity were not analyzed in detail and were combined into one residual group.

## Results

### Study populations in 1987 and 2001

The study population in 1987 consisted of 86,577 children yielding 21,644 person years. These children presented a total of 9,271 contacts with skin problems which contributed to 6,870 episodes; 75.4% of these episodes resulted in a single contact with the GP. In 2001 there were 88,307 children yielding 82,053 person-years. These children presented a total of 29,637 contacts with skin problems that contributed to 23,586 episodes; 76.6% of these episodes consisted of only one contact with the GP.

### All episodes of skin disease

Table 1 shows the distribution and the change in incidence rates of between 1987 and 2001 of skin diseases in general practice, stratified for several background characteristics. Compared to 1987, in 2001 the overall incidence rate of skin diseases combined had decreased significantly from 317.4, CI: [309.9–325.0] to 287.5, CI: [283.8–291.2] per 1000 person years. The incidence rate of skin diseases presented to the GP increased among infants (children under one year); in all other age categories except age category '1–4 years' the incidence rates decreased.

In 2001, girls presented significantly more skin diseases to the GP than boys. There was a similar geographic gradient in both surveys: in the southern part of the Netherlands children presented more often skin diseases to the GP compared to the northern part. In 2001, the incidence rate of skin diseases presented to the GP increased in rural areas whereas it decreased in suburban areas. In the big cities the incidence rate remained stable. In both surveys the seasonal peak was in spring. In 2001 children with parents in SES class I, II, IV, V showed a decrease of the incidence rate of skin diseases presented to the GP whereas the incidence rate in class III (non-manual low and farmers) remained stable compared to 1987. In both surveys the incidence rates of skin diseases in general practice were higher in lower SES classes.

In 2001 children of non-Western immigrants visited the GP more often with skin diseases than children of natives and western immigrants combined.

Table 2 shows the incidence rates of skin diseases in general practice for the distinct ICPC codes. In 2001 incidence rates are shown for separate age categories and compared with the crude incidence rate in 1987. In both surveys warts, impetigo, dermatophytosis, contact dermatitis, atopic dermatitis and injuries of the skin were the most frequent skin diseases, accounting for about 57% of the total skin-related morbidity presented to the GP. Although, in general practice the incidence rate of warts decreased by 23% in 2001, it remained the most frequent skin disease in children in both surveys. In 2001, in general practice the incidence rate of impetigo, dermatophytosis and atopic dermatitis increased whereas the incidence rate of the most viral skin infections decreased. Also contact dermatitis and several types of skin injuries showed a decreased incidence rate in general practice. Most of the specific skin diseases (e.g. dermatophytosis, moniliasis/candidiasis, contact dermatitis, atopic dermatitis and diaper rash) showed the highest incidence rate among infants in general practice.

## Discussion

These two large and representative surveys give a comprehensive assessment of the dermatological morbidity in children encountered in Dutch general practice, and enabled us to estimate current incidence rates for all skin diseases.

The overall incidence rate of skin diseases presented to the GP decreased by 9.4%, which is surprising given the decreased overall consultation rate by children as reported elsewhere [5]. According to the decrease of the overall consultation rate by 22% we expected a lower incidence rate of skin diseases in 2001 in general practice.

In infants, the incidence rate of skin diseases presented to the GP has increased in 2001, especially of atopic dermatitis and moniliasis/candidiasis [table 2]. This increase is in accordance with previous studies showing an increase of atopic dermatitis in the general population [8,9].

Girls visited the GP more often concerning skin problems which is in accordance with previous studies [1,2,14]. Probably this difference is based on aesthetic reasons.

Between 1987 and 2001, the incidence rate of skin diseases in general practice increased in rural areas and decreased in suburban areas. It seems plausible that this increase could partly be explained by the increased incidence of bacterial skin infections in our data. It is suggested elsewhere that children in rural areas are more exposed to infectious pathogens due to the larger number of animals and farms [15]. Actually, with the decreasing consultation rate we would expect also a decrease of the incidence rate of skin diseases in general practice in urban areas but this did not change between 1987 and 2001. Probably children in urban areas are suffering more from skin diseases. This is in accordance with the 'the pollution hypothesis' meaning that children in urban areas have a higher chance in developing atopic diseases [15,16]. Crowding in urban areas could be a potential factor in spreading infectious skin diseases.

According to table 1 there is a regional variation in the incidence rates of skin diseases in general practice. The highest incidence rates of skin diseases encountered in practices in the south is a striking observation, especially for a small country like the Netherlands. Of the six most frequent skin diseases impetigo (S84) had a geographical gradient with a two fold higher incidence rate in the south compared to the north. Also for non-dermatological conditions we found a significantly higher consultation rate in the south of the Netherlands in both surveys. Practice characteristics seem not to play an important role.

In 2001, children of non-Western immigrants consulted their GP more often with skin diseases. The significantly higher consultation rate and the proportional increase of non-Western children from 7.4% to 9.7% in the Dutch childhood population might explain these differences [5,6]. Probably, non-Western children suffer more from skin diseases; a previous study reported that non-Western immigrants in the Netherlands more often felt unhealthy [13].

We found the highest incidence rate of skin diseases in general practice in the lower SES classes, which is consistent with previous studies [17,18]. The most striking finding is that the incidence rate of skin diseases in general practice remained stable in SES class III [table 1]. In all other SES classes the incidence rate have decreased between 1987 and 2001 which is in accordance with the decreased consultation rate in general practice [5].

Furthermore, from table 2 it becomes clear that the incidence rates of some skin diseases presented to the GP have increased while others have decreased substantially. The increased incidence rate of infectious (bacterial and fungal) and allergic skin diseases in our data is compatible with reported trends [5,8,9,14]. The increasing use of day-care and after-school facilities in the Netherlands might explain the increase of infectious skin diseases in general practice [15]. There are suggestions that the increasing use of topical antibiotics resulted in more resistant bacterial strains, which could have resulted in a rise of the incidence of bacterial skin infections in the population. The striking decrease in incidence rate of most viral skin infections in our data was in parallel with the decreasing consultation rates. In 2001, infectious skin diseases represent a substantial part (45%) of the total skin morbidity presented to the GP which is in accordance with previous studies [3,4]. Skin injuries and allergic skin diseases contributed about 15% and 18% respectively.

This study had some limitations. There were small differences in the design of the two national surveys, which might disturb the comparability of data. Some of the differences in occurrence may be explained by the fact that ICPC coding was not performed equally in both surveys: in 1987 clerks coded diagnoses afterwards, whereas in 2001 the GPs coded the diagnoses themselves during the consultation. We assume that coding by clerks more often led to a diagnosis-specific ICPC code. In the present study the accuracy of diagnoses made by the GPs could be a subject of debate. In our analysis we assumed that the diagnoses made by the GPs were correct. In 2001 the participating GPs were trained in coding the diagnoses correctly using ICPC codes. Overall these trained GPs classified diagnoses correctly in about 81% of the test cases [19].

## Conclusion

The overall incidence rate of all skin diseases combined in general practice decreased whereas the incidence rates of bacterial, mycotic and atopic skin diseases increased. On these topics more detailed epidemiological data and population-based prevalence studies are needed.

## Competing interests

All author(s) declare that they have no competing interests.

## Authors' contributions

RSAM and JCvdW designed the study. RSAM carried out the analyses and drafted the paper. All authors commented on draft versions and approved the final manuscript.

## Funding

The Dutch ministry of Health, Welfare and Sports mainly funded the surveys directly or indirectly. In addition, the "Stichting Centraal Fonds RVVZ" contributed financially to the second survey. The analysis reported in this paper was made possible through internal funding of the department of General Practice, Erasmus MC-University Medical Center Rotterdam.

## Pre-publication history

The pre-publication history for this paper can be accessed here:



## Figures and Tables

**Table 1:** Incidence rates per 1000 person years of all new episodes of all skin diseases combined in general practice in 1987 and 2001.

	1987		2001		
	Incidence Rates	95% Confidence Intervals	Incidence Rates	95% Confidence Intervals	P-value

Age Categories					
< 1 year	352.1	321.0 – 385.5	460.8	441.0 – 481.3	<0.001
1–4 years	328.9	312.2 – 346.2	320.0	311.8 – 328.3	0.35
5 – 9 years	340.3	325.4 – 355.7	273.7	267.1 – 280.5	<0.001
10 – 14 years	276.2	262.8 – 290.1	235.3	229.1 – 241.6	<0.001
15 – 17 years	319.0	302.5 – 336.3	266.9	258.3 – 275.7	<0.001
Gender					
Male	315.9	305.5 – 326.5	279.2	274.2 – 284.3	<0.001
Female	319.0	308.3 – 330.0	296.2	290.8 – 301.5	<0.002
Urbanization					
< 30,000	307.8	295.3 – 320.6	324.3	318.4 – 330.4	0.02
30,000 – 50,000	315.4	303.1 – 328.1	253.9	246.0 – 261.9	<0.001
> 50,000	349.9	332.6 – 367.9	258.1	251.9 – 264.3	<0.001
Big Cities	289.1	265.5 – 314.2	285.8	271.7 – 300.5	0.82
Region					
Northern	303.3	281.8 – 326.0	253.6	244.6 – 262.8	<0.001
Central	310.2	301.0 – 319.6	282.4	277.6 – 287.3	<0.001
Southern	342.7	327.1 – 358.8	314.6	307.4 – 321.8	0.001
Season					
Winter	305.8	290.4 – 321.8	279.4	272.2 – 286.8	0.003
Spring	336.6	322.5 – 351.1	294.5	287.1 – 302.1	<0.001
Summer	319.3	302.9 – 336.4	292.5	285.1 – 300.0	0.004
Autumn	304.9	291.1 – 319.2	272.7	265.8 – 279.8	<0.001
SES					
Class I	305.0	289.3 – 321.2	265.2	258.6 – 271.9	<0.001
Class II	325.2	304.1 – 347.4	278.8	270.8 – 287.0	<0.001
Class III	264.2	232.2 – 299.4	299.0	284.4 – 314.0	0.06
Class IV	340.9	322.1 – 360.6	299.5	283.6 – 316.1	0.001
Class V	336.4	315.8 – 358.0	283.2	270.4 – 296.5	<0.001
Ethnicity					
Natives – Western Immigrants	315.7	308.1 – 323.5	275.1	270.7 – 279.6	<0.001
Non-Western Immigrants	346.1	313.6 – 380.9	295.6	281.6 – 310.0	0.01
Total	317.4	309.9 – 325.0	287.5	283.8 – 291.2	<0.001

**Table 2:** Incidence rates per 1000 person years of all new episodes of skin diseases according to ICPC codes in Dutch general practice in 1987 and 2001.

					2001						1987
					< 1 year	1–4 years	5–9 years	10–14 years	15–17 years	0–17 years	0–17 years

	ICPC		N	%							

Bacterial infections	S84	Impetigo	1684	7.1	18.6	29.4	27.7	13.4	7.3	20.5	16.5
	S11	Localized skin infections	726	3.1	10.2	9.0	9.1	7.4	9.4	8.8	4.5
	S09	Infected finger/toe/paronychia	455	1.9	7.7	7.3	5.3	5.1	3.2	5.5	7.7
	S10	Carbuncle/Cellulitis	196	0.8	0.9	2.0	2.8	2.0	3.1	2.4	6.1
	S76	Erysipelas/Erythrasma	153	0.6	2.3	2.4	1.4	1.5	2.3	1.9	3.2
Viral Infections	S03	Warts	2816	11.9	0.7	13.7	54.8	38.7	27.4	34.3	44.8
	S95	Mollusca contagiosa	777	3.3	2.3	18.1	17.0	1.4	0.3	9.5	10.8
	S71	Herpes Simplex skin/lip	148	0.6	1.8	2.6	1.2	1.8	1.8	1.8	2.9
	S70	Herpes Zoster	134	0.6	0.0	1.6	1.4	2.1	1.5	1.6	2.0
Fungal Infections	S74	Dermatophytosis	2085	8.8	41.0	24.7	20.3	24.6	29.6	25.4	20.8
	S75	Moniliasis/candidiasis	801	3.4	112.9	12.5	1.4	1.0	1.2	9.8	4.9
Parasitic Infestations	S12	Insect bite	577	2.4	9.3	11.8	6.9	4.0	4.7	7.0	6.7
Dermatitis	S88	Contact dermatitis/other eczema	1882	8.0	31.1	24.6	18.9	19.8	28.5	22.9	33.2
	S87	Atopic dermatitis	1353	5.7	90.9	26.0	9.5	7.1	6.5	16.5	11.3
	S86	Seborrhoeic dermatitis	416	1.8	24.7	3.3	2.4	5.2	5.3	5.1	3.4
	S89	Diaper rash	406	1.7	48.5	10.3	0.2	0.0	0.0	4.9	10.5
	S90	Pityriasis rosea	124	0.5	0.2	0.5	1.1	2.1	2.8	1.5	1.2
Neoplasms	S82	Naevus/mole	479	2.0	1.4	1.3	4.5	7.9	11.9	5.8	3.5
	S04	Local swelling/mass	372	1.6	8.2	4.6	3.9	4.0	5.0	4.5	2.0
	S79	Other benign neoplasms	203	0.9	1.8	1.0	1.6	3.1	5.1	2.5	3.8
	S93	Sebaceous cyst	184	0.8	1.4	1.3	1.4	2.2	5.2	2.2	3.9
Injuries	S18	Laceration/cuts	1668	7.1	7.9	30.8	21.8	15.0	15.1	20.3	23.4
	S16	Bruises/Contusions	713	3.0	6.6	7.9	7.2	10.0	10.2	8.7	7.8
	S17	Abrasion/scratch/blister	440	1.9	2.3	5.8	5.3	4.4	7.1	5.4	6.0
	S14	Burns/scalds	255	1.1	5.0	4.9	1.4	2.2	4.4	3.1	3.4
	S13	Human / animal bite	169	0.7	1.6	2.2	2.0	2.0	2.1	2.1	2.5
	S15	Foreign body in skin	131	0.6	0.5	1.6	2.0	1.5	1.3	1.6	1.9
	S19	Other injuries to skin	165	0.7	1.6	2.5	2.4	1.6	1.4	2.0	11.8
Others	S98	Urticaria	673	2.9	6.3	12.0	9.2	5.4	6.2	8.2	7.4
	S96	Acne	506	2.1	0.2	0.1	0.3	9.9	19.7	6.2	9.9
	S06	Erythema/rash	437	1.9	22.4	7.9	3.7	2.5	3.7	5.3	4.2
	S94	Ingrown toenail/other diseases of nail	430	1.8	0.9	1.5	1.6	9.1	11.0	5.2	4.9
	S21	Symptoms/complaints of skin texture	349	1.5	15.6	5.5	3.3	2.3	3.5	4.3	2.3
	S02	Pruritis/skin itching	310	1.3	3.2	4.8	3.7	2.7	4.3	3.8	2.3
	S29	Other symptoms/complaints skin	207	0.9	8.4	2.0	1.4	2.2	3.5	2.5	1.5
	S99	Other diseases of skin/subcutaneous tissue	184	0.8	4.1	1.6	1.5	2.3	3.6	2.2	5.8
	S22	Symptoms/complaints of nails	126	0.5	1.4	1.6	1.2	1.7	1.6	1.5	1.3
Residuals			852	3.6						10.4	16.3
Total			23586	100						287.5	317.4

ICPC = International Classification of Primary Care

N = number of incident episodes of skin diseases

% = percentage of new episodes of skin diseases with a distinct ICPC
